# Collargolum in Surgery

**Published:** 1907-03

**Authors:** 


### Collargolum in Surgery.

In his work on the Russo-Japanese war, von Oettingen describes a new method of wound dressing which he employed in thousands of cases. He and his colleagues in the field hospital never attempted to cleanse the wounds; they painted the surrounding area with a solution of mastic, placed a collargol tablet into the lesion, covered it with a sterile cotton pad, and applied a bandage. The mastic "arrested" the bacteria on the skin and prevented their migration into the wounds, especially because the dressing was immovable. The tablet immediately dissolved and penetrated into the crevices, whence the silver was absorbed. Collargolum has an indubitable antiseptic action and does not coagulate blood serum, so that the secretion flows off freely till the wound is clean; then a scab forms which soon drops off from the cicatrix.

He also used a 3 per cent. collargol-lactose dusting powder, which is even cheaper than iodoform, as well as 1:500 to 1:1000

solutions for irrigating facial sinuous wounds, the bladder, and the urethra. At laparotomies a 1:100 to 1:500 solution was poured into the cavities. Collargol is a surgical antiseptic to which no objection can be raised; it is nontoxic, nonirritant and obviates the need of working in phenol or lysol odors. The bichloride in the first aid packages should be replaced by collargolum, this giving a nontoxic antiseptic bandage kit.

